# ﻿A new species of *Dipsas* (Serpentes, Dipsadidae) from central Panama

**DOI:** 10.3897/zookeys.1145.96616

**Published:** 2023-02-03

**Authors:** Julie M. Ray, Paola Sánchez-Martínez, Abel Batista, Daniel G. Mulcahy, Coleman M. Sheehy III, Eric N. Smith, R. Alexander Pyron, Alejandro Arteaga

**Affiliations:** 1 Department of Biology, University of Nevada - Reno, Reno, Nevada 89557, USA University of Nevada-Reno Reno United States of America; 2 Museu de Zoologia da Universidade de São Paulo, São Paulo, Brasil. Av. Nazaré 481, Ipiranga, 04263-000, São Paulo, Brazil Museu de Zoologia da Universidade de São Paulo São Paulo Brazil; 3 Instituto Interdisciplinario de investigación e innovación, Universidad Autónoma de Chiriquí (UNACHI), David, Chiriquí, Panama Universidad Autónoma de Chiriquí (UNACHI) David Panama; 4 Fundación Los Naturalistas, Boquete, Chiriquí, Panama Fundación Los Naturalistas Boquete Panama; 5 Sistema Nacional de Investigación (SNI), SENACYT, Panama Sistema Nacional de Investigación (SNI) Senacyt Panama; 6 Department of Vertebrate Zoology, National Museum of Natural History, Smithsonian Institution, Washington, D.C. 20560, USA National Museum of Natural History, Smithsonian Institution Washington United States of America; 7 Division of Herpetology, Florida Museum of Natural History, University of Florida, Gainesville, Florida 32611, USA University of Florida Gainesville United States of America; 8 Amphibian and Reptile Diversity Research Center, Department of Biology, University of Texas at Arlington, Arlington, Texas 76019, USA University of Texas at Arlington Arlington United States of America; 9 Department of Biological Sciences, The George Washington University, 2029 G St. NW, Washington DC, 20052, USA The George Washington University Washington United States of America; 10 Biodiversity Field Lab (BioFL), Khamai Foundation, Quito, Ecuador Biodiversity Field Lab (BioFL), Khamai Foundation Quito Ecuador

**Keywords:** Dipsadini, *Dipsastemporalis*, new species, phylogeny, snail-eating snake, systematics

## Abstract

A new species of *Dipsas* Laurenti, 1768, from Central Panama is described based on molecular analyses, hemipenial morphology, and external characters. This is the sixth species of *Dipsas* to be described for the country; the snake has been suspected to exist since 1977 and has not been thoroughly studied until now. Additionally, morphological comparations including scale counts are done with other species within the genus, and the current geographic distribution of *Dipsastemporalis* (Werner, 1909), the sister species, is updated. Finally, a key to the species of *Dipsas* currently known from Middle America is presented.

## Abstract (es)

Describimos una nueva especies de *Dipsas* Laurenti, 1768 de la región central de Panamá en base a análisis moleculares, morfología hemipenial y caracteres de morfología externa. Esta es la sexta especie del género *Dipsas* descrita para el país. Se sospechaba su existencia desde 1977 pero no había sido estudiada exhaustivamente hasta ahora. Adicionalmente, presentamos comparaciones morfológicas (incluyendo lepidosis) con otras especies del género y actualizamos la distribución geográfica de su especie hermana *Dipsastemporalis* (Werner, 1909). Finalmente, presentamos una clave para las especies de *Dipsas* distribuidas en Centroamérica.

## ﻿Introduction

The Neotropical snake genus *Dipsas* Laurenti, 1768, belongs to the tribe Dipsadini, a group of primarily arboreal snakes that includes the genera *Dipsas*, *Plesiodipsas*Harvey et al., 2008, *Sibon* Fitzinger, 1826, and *Tropidodipsas* Günther, 1858 (Harvey et al. 2008; Zaher et al. 2009; Grazziotin et al. 2012; Arteaga et al. 2018). The “snail-eating” snakes (Mertens 1952; Peters 1956) or “snail-suckers” (Peters 1956) are part of a larger group of neotropical snakes called the “goo-eaters” (Cadle and Greene 1993) because of their proclivity for feeding on soft and often slimy invertebrates. According to Cadle and Greene (1993), the “goo-eaters” also include the mainly earthworm- and slug-eating species in the genera *Adelphicos* Jan, 1862, *Atractus* Wagler, 1828, *Geophis* Wagler, 1830, and *Ninia* Baird & Girard, 1853, and possibly also *Chersodromus* Reinhardt, 1860 and *Cryophis* Bogert & Duellman, 1963 (see Sheehy 2012); though, *Cryophis* is also known for preying on salamanders (Mulcahy 2007). The discovery of a broader diet for some snail-eating snakes of the genera *Sibon* and *Dipsas* to include additional invertebrates and anuran eggs further refined our understanding of the diet of these snakes (Ryan and Lips 2004; Montgomery et al. 2007; Ray et al. 2012).

The genus *Dipsas* currently contains 53 small- to moderately-sized species that can be distinguished from the other genera of the tribe by external features, such as body often strongly compressed (in arboreal taxa), head distinct from neck, usually more than 10 infralabials, vertebral scale row usually enlarged, preoculars 0–2, supralabials and infralabials not notably enlarged, mental groove very weak to absent, and often two or more pairs of infralabials in contact behind mental (Peters 1960; Harvey and Embert 2008; Uetz et al. 2022). Harvey and Embert (2008) also describe internal characteristics, including a well-developed tracheal lung and characteristics of the hemipenes. Species of *Dipsas* are Neotropical and range from central Mexico to southern South America (Peters 1960; Solórzano 2004; Ray 2009), and five species are currently recognized in Panamanian territory: *D.articulata* Cope, 1868, *D.nicholsi* (Dunn, 1933), *D.temporalis* (Werner, 1909), *D.tenuissima* Taylor, 1954, and *D.viguieri* (Bocourt, 1884). Detailed reviews of Panamanian *Dipsas* are provided by Peters (1960), Savage (2002), Cadle and Myers (2003), and Ray (2017). Of these, *D.tenuissima* is at the southern and easternmost extent of its range and *D.viguieri* is at the northern and westernmost extent of its range in Panama (Ray 2017). Based on current information, *D.nicholsi* is endemic to the country, with most records found east of the Panama Canal and one record west of it (Myers et al. 2007). *Dipsastemporalis* was, until now, one of the most widespread of the species of *Dipsas* in Panama (Ray 2017).

The most complete, recent taxonomic review of the genus was by Peters (1960), who principally used color pattern to recognize species. This has led to several remaining taxonomic issues. The availability of new material has resulted in species and groups of species within the genus being revised frequently in subsequent years (Cadle and Myers 2003; Passos et al. 2004, 2005; Cadle 2005; Harvey 2008; Harvey and Embert 2008; Sheehy 2012; Arteaga et al. 2018). Phylogenetic relationships among species of *Dipsas* and closely related genera remain unclear, since most phylogenetic studies published regarding snake systematics (Zaher et al. 2009; Vidal et al. 2010; Grazziotin et al. 2012; Pyron et al. 2013; Figueroa et al. 2016) have not sampled a sufficient set of species in these genera. However, all these studies corroborated paraphyly of the genus *Dipsas* with respect to *Sibynomorphus* (see Sheehy 2012). A recent study focused on the systematics of South American *Dipsas* and *Sibon* described several new species, and synonymized *Sibynomorphus* with *Dipsas* (Arteaga et al. 2018).

Between 1997 and 2015, one of us (JMR) regularly studied reptiles and amphibians in Parque Nacional General de División Omar Torrijos Herrera (PNGDOTH), near the community of El Copé de La Pintada, Coclé Province, Republic of Panama. In 1977, before the area was established as a national park, it was visited by the late Charles W. Myers, who suggested that at least one undescribed species of *Dipsas* occurred at the site (Myers et al. 2007). More recently, other researchers have agreed with that assessment (Ray et al. 2012). After examination of specimens collected in 2006–2009 and 2011 and after analysis of molecular data, including the updated phylogeny constructed for this paper, we confirm the existence of at least one new species of *Dipsas* at this site, which we herein describe. Additionally, we have confirmed the presence of this species at other sites. We also confirm that *Dipsastemporalis*, the species to which this snake was believed to belong, is still found in Panama; thus, we update the range of *D.temporalis*. Finally, we provide a key to the Central American species of the genus *Dipsas*.

## ﻿Materials and methods

### ﻿Ethics statement

This study was carried out in strict accordance with the guidelines for use of live amphibians and reptiles in field research (Beaupre et al. 2004) compiled by the American Society of Ichthyologists and Herpetologists (**ASIH**), the Herpetologists’ League (**HL**), and the Society for the Study of Amphibians and Reptiles (**SSAR**). All procedures with animals (see below) were reviewed by the Ministerio del Ambiente, Agua y Transición Ecológica (**MAATE**) in Ecuador and by UNARGEN-Ministerio de Ambiente in Panamá, and specifically approved as part of obtaining the following field permits for research and collection: MAE-DNB-CM-2018-0105 and MAATE-DBI-CM-2022-0245 (granted to Universidad San Francisco de Quito) and SC/A-8-09, SC/A-28-09, SC/A-37-11, SC/A-33-12, SE/A-60-16, and SE/A-33-18 (granted to Museo Herpetológico de Chiriquí). Specimens were euthanized with 20% benzocaine, fixed in 10% formalin or 90% ethanol, and stored in 70% ethanol. Museum vouchers were deposited at the Smithsonian National Museum (**USNM**), Museo Herpetológico de Chiriquí (**MHCH**), the Senckenberg Forschungsinstitut Frankfurt (**SMF**), and at Museo de Zoología de la Universidad San Francisco de Quito (**ZSFQ**).

### ﻿Common names

Criteria for common name designation are as proposed by Caramaschi et al. (2006) and Coloma and Guayasamin (2011–2017), reviewed by Arteaga et al. (2019). These are as follows (in order of importance): (i) the etymological intention (implicit or explicit) that the authors used when naming the species (specific epithet); (ii) a common name that is already widely used in the scientific literature; (iii) a common name that has an important ancestral or cultural meaning; (iv) a common name based on any distinctive aspect of the species (distribution, morphology, behavior, etc.).

### ﻿Material examined

We examined 31 specimens suspected to be a new species from 15 locations in Panama. Of these, we examined 23 specimens collected at Parque Nacional General de División Omar Torrijos Herrera (**PNGDOTH**), located 7.5 km north of the community of El Copé de La Pintada, Coclé Province, Republic of Panama (8.670383, -80.592343, 763 m a.s.l.) between 650 and 850 m. Specimens from eight other species of *Dipsas* also were examined for comparison purposes (Appendix 1).

We gathered additional data for the Central American species of *Dipsas* from Peters (1960), Savage (2002), Cadle and Myers (2003), Solórzano (2004), and Ray (2017). We follow Dowling (1951) for the method of counting ventrals and subcaudals and Savage (1973) for the terminology of scales in the loreal region of the head. We follow Peters (1960) and Harvey and Embert (2008) for terminology for cephalic shields. Sex was determined by probe or by subcaudal incision unless hemipenes were everted. Head and scale measurements were made to the nearest 0.1 mm using digital calipers held under a dissecting microscope. Snout-vent length and tail length measurements were taken to the nearest 1.0 mm using a squeeze box (Quinn and Parker 1976) or tape measure.

Terminology for measurements is abbreviated as: snout-vent length, **SVL**; tail length, **TL**; total length, **TOL**; head length, **HL**; jaw length, **JL**; and head width, **HW**. Eye length equals the horizontal distance across eye at widest point. Scale dimensions were measured at the longest or widest points along the longitudinal or perpendicular axis of the body, respectively. Drawings of the head were made using digital photography and a dissecting microscope by Shannon Christensen. Hemipenial preparation follows Zaher (1999) and Zaher and Prudente (2003). Once prepared, the hemipenes were stained with alizarin in 70% ethanol to facilitate the visualization of calcified structures (Harvey and Embert 2008; Nunes et al. 2012).

### ﻿Molecular phylogenetics

A subset of molecular data is presented here for 19 species of *Dipsas* (Appendix 3), taken from the thesis of CMS (Sheehy 2012), which included 175 total taxa representing most other genera in the subfamily Dipsadinae. Five loci were used: (1) a 714 base pair fragment of the mitochondrial NADH dehydrogenase subunit 4 (ND4), (2) a 199 base pair fragment of tRNAs His, Ser and Leu, (3) a 1071 base pair fragment of the mitochondrial cytochrome-b gene (cyt-b), (4) a 525 base pair fragment of the nuclear protein-coding neurotrophin-3 (NT3) gene, and (5) a 732 base pair fragment of the nuclear protein-coding dynein, axonemal, heavy chain 3 (DNAH3) gene (see Appendix 2 for primers used). Genomic DNA was isolated from tissues using a Qiagen DNeasy kit (Qiagen, Valencia, California, USA). All amplification reactions used GoTaq Green Master Mix, 2X (Promega Corporation, Madison, Wisconsin, USA). Thermal cycling followed standard protocols and are detailed in Sheehy (2012). Successfully amplified PCR products were prepared for sequencing by using the ExoSAP-IT kit (United States Biochemical). A BigDye Terminator Cycle Sequencing Kit (Applied Biosystems Inc.) was used for sequencing following the manufacturer’s protocol and using PCR primers. The sequenced products were precipitated using an ethanol/sodium acetate method and rehydrated in HPLC purified formamide (HIDI). The sample was then analyzed on an ABI PRISM 3100xl Genetic Analyzer in the Genomics Core Facility at the University of Texas at Arlington, USA.

Alignments were constructed using the program Sequencher 4.8 (Gene Codes, Ann Arbor, Michigan, USA), and edited by eye using the program MacClade 4.08 (Maddison and Maddison 2005). The tRNAs were aligned using an annotated mitochondrial genome for *Sibonnebulatus* (GenBank accession number EU728583; Mulcahy and Macey 2009) as a template sequence.

Phylogenetic analyses were conducted using Maximum Likelihood (ML) and Bayesian Index (BI) on the data matrix consisting of 194 taxa and up to 3241 base pairs. Various models of molecular evolution were tested using the software package MEGA 5 (Tamura et al. 2011) on the complete alignment partitioned by gene fragment (seven partitions: ND4, cytb, tRNA His, tRNA Ser, tRNA Leu, NT3, and DNAH3). The model test results identified GTR+I+G and GTR+G as among the best-fit models of nucleotide substitution for each gene fragment based on corrected Akaike Information Criterion (AICc), although they did not always receive the best scores. The ML analyses employing the rapid bootstrapping algorithm were conducted using the program RAxML 7.3.0 (Stamatakis 2006) on the CIPRIS Science Gateway server v. 3.2 (Miller et al. 2010) using the model GTR+G instead of GTR+I+G because the 25 discrete rate categories appear to better estimate invariant sites (Stamatakis 2006). The multiple alignment was partitioned by gene region (five partitions: ND4, cytb, tRNAs, NT3, DNAH3), which allowed RAxML to calculate and apply the most appropriate gamma distribution parameter to each partition separately. Nodal support for ML was provided by rapid bootstrapping (1000 pseudoreplicates), with bootstrap values ≥ 0.70 considered strong support (Hillis and Bull 1993).

Bayesian analyses were conducted with the computer program MrBayes (Huelsenbeck and Ronquist 2001) on a partitioned alignment using the reversible-jump Markov chain Monte Carlo algorithm (mixed model), which avoids the risk of acquiring misleadingly high posterior probabilities at the nodes of hard or nearly hard polytomies due to their arbitrary resolution (Lewis et al. 2005). Each of the four protein coding genes in the alignment was partitioned by codon position with one partition including the first and second positions and another including the third position for a total of nine partition schemes (the three tRNAs were not partitioned).

Two independent runs were conducted simultaneously with four Markov chains (three heated and one cold) per run, and average standard deviation of the split frequencies below 0.01 was considered acceptable. Stationarity was determined to be reached visually using Tracer v. 1.5 (Rambaut and Drummond 2009). The analysis ran for 17,000,000 generations while sampling trees every 1000 generations. Stationarity was reached after approximately 11,500,000 generations, after which the standard deviation of the split frequencies dropped to 0.008. Therefore, we sampled the resulting 5000 trees from the last five million generations (12–17 million generations), which should be a good representation of the posterior distribution of trees. The initial 12 million generations were discarded as burn-in, and a 50% majority rule consensus tree with estimates of Bayesian support was constructed using the remaining sampled trees. Posterior probabilities (PP) provided nodal support for Bayesian analyses, with PP values ≥0.95 considered strong support (Alfaro et al. 2003; Huelsenbeck and Rannala 2004; Mulcahy et al. 2011).

### ﻿Distribution maps and ecological niche models

We present ranges of occurrence for two species of *Dipsas*, *D.temporalis* and a new species herein described. Presence localities are derived from museum vouchers (Appendix 1), photographic records (iNaturalist), and the literature. For each species, a binary environmental niche model (ENM) accompanies the dot maps. These models estimate potential areas of distribution based on observed presences and a set of environmental predictors (Elith and Leathwick 2009). To delimit the occupancy areas and the potential species distribution, we used the BAM diagram proposal (Soberón and Peterson 2005; Peterson et al. 2011). To create the models, we used presence localities as described above, 19 bioclimatic variables from Worldclim 1.4 (Hijmans et al. 2005), and Maxent 3.4.1k, an algorithm based on the principle of maximum entropy (Phillips et al. 2006; Elith et al. 2011; Renner and Warton 2013).

For the first explorative exercise, we used the 19 climate layers from the WorldClim project and assessed which variables were the most important for the model, according to the Jackknife test calculated in MaxEnt (Royle et al. 2012). Correlated environmental variables (r < 0.8) were identified using the PEARSON correlation test of PAST 3. In a second modelling exercise, we used the locality records for each species and the variables identified in the first approach to generate the species distribution. 5,000 iterations were specified to the program with clamping and no extrapolation. All other parameters in MaxEnt were maintained at default settings. To create the binary environmental niche models, suitable areas were distinguished from unsuitable areas by setting a minimum training presence threshold value. The logistic format was used to obtain the values for habitat suitability (continuous probability from 0 to 1), which were subsequently converted to binary presence-absence values on the basis of the established threshold value, defined herein as the minimum training presence. The convergence threshold was set to 10^-5^, maximum iterations to 500, and the regularization parameter to “auto.”

## ﻿Results

### ﻿Systematics

**Figure 1.**
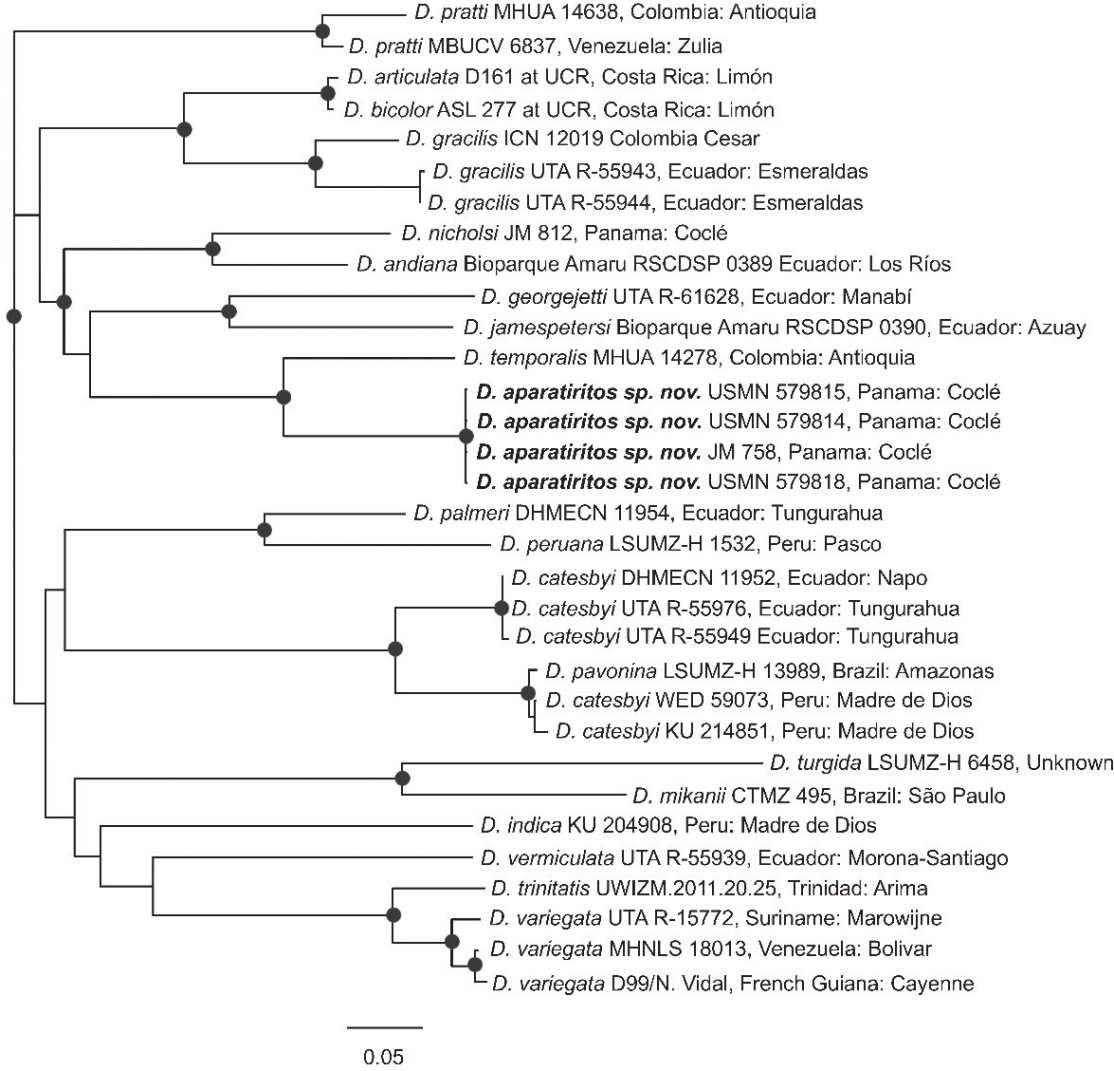
Phylogeny of 20 species of *Dipsas* using the best ML tree. Black circles denote strong nodal support (≥ 0.95 PP and ≥ 0.70 ML bootstrap). See Sheehy (2012) for further details on the outgroup taxa.

The ML and Bayesian analyses were largely congruent, particularly with respect to the well-supported clades. The ML phylogeny of a well-supported clade containing most species of *Dipsas* sampled (except *“D.” gaigeae*; see Sheehy 2012) is here presented, with Bayesian posterior-probabilities superimposed on well-supported nodes (Fig. 1). The specimens from PNGDOTH formed a clearly divergent, strongly supported lineage separate from the other Central American species and is sister to *Dipsastemporalis*, to which it differs by ~ 7% (uncorrected pairwise-distance) for the ND4 locus. Based on this genetic distinctiveness, along with discontinuous morphological variation in scalation and unique hemipenes morphology (see below), we determine that it does, indeed, represent a new species as previously hypothesized.

**Figure 2.**
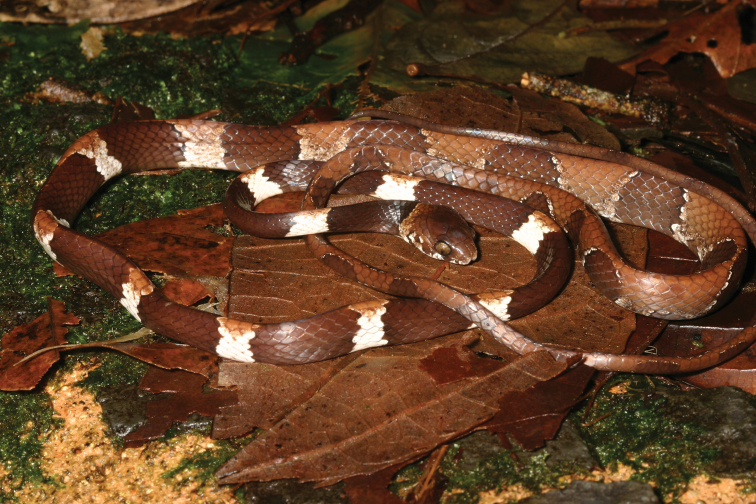
Live individual of *Dipsasaparatiritos* sp. nov. in Parque Nacional General de División Omar Torrijos Herrera photographed in the wild and not collected. Photography by Kevin Enge.

#### ﻿ Dipsas aparatiritos sp. nov.

Taxon classificationAnimaliaSquamataDipsadidae

DCA0A709-F325-59FE-A2F0-484868C13C77

https://zoobank.org/E96CAB59-FBB7-451B-9D11-4372182F9809

**Figure 3.**
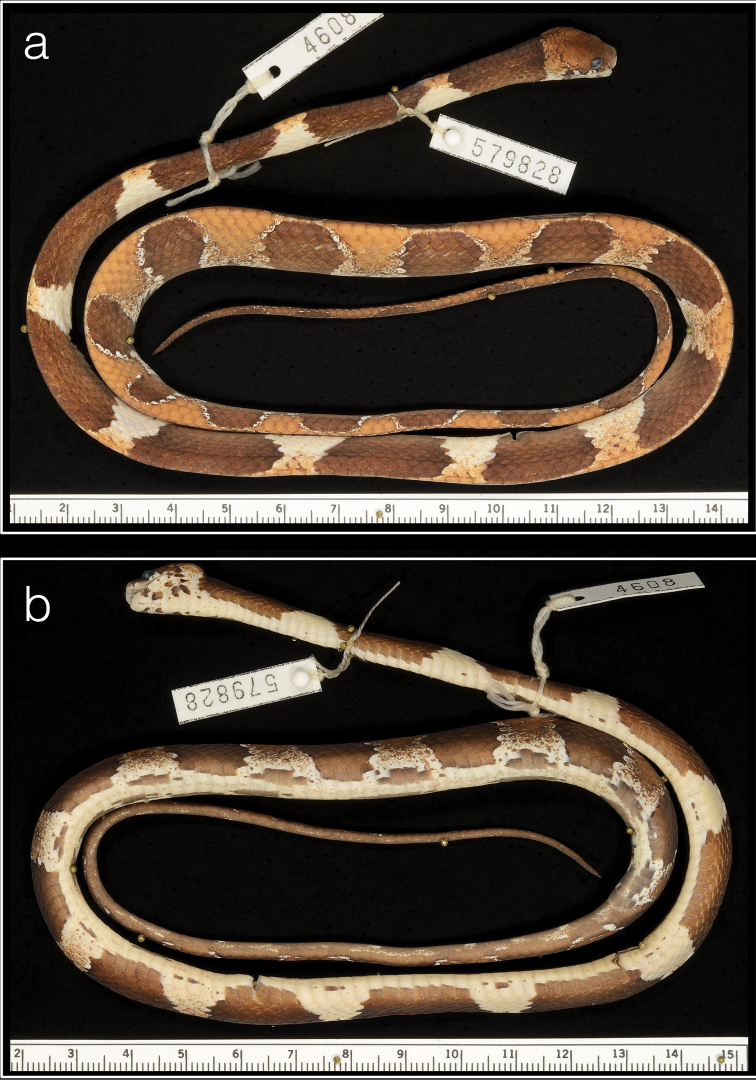
Holotype (USNM 579828) of *Dipsasaparatiritos* sp. nov. showing **a** dorsum and **b** venter. Ruler units in cm. Photographs by James Poindexter.

**Figure 4.**
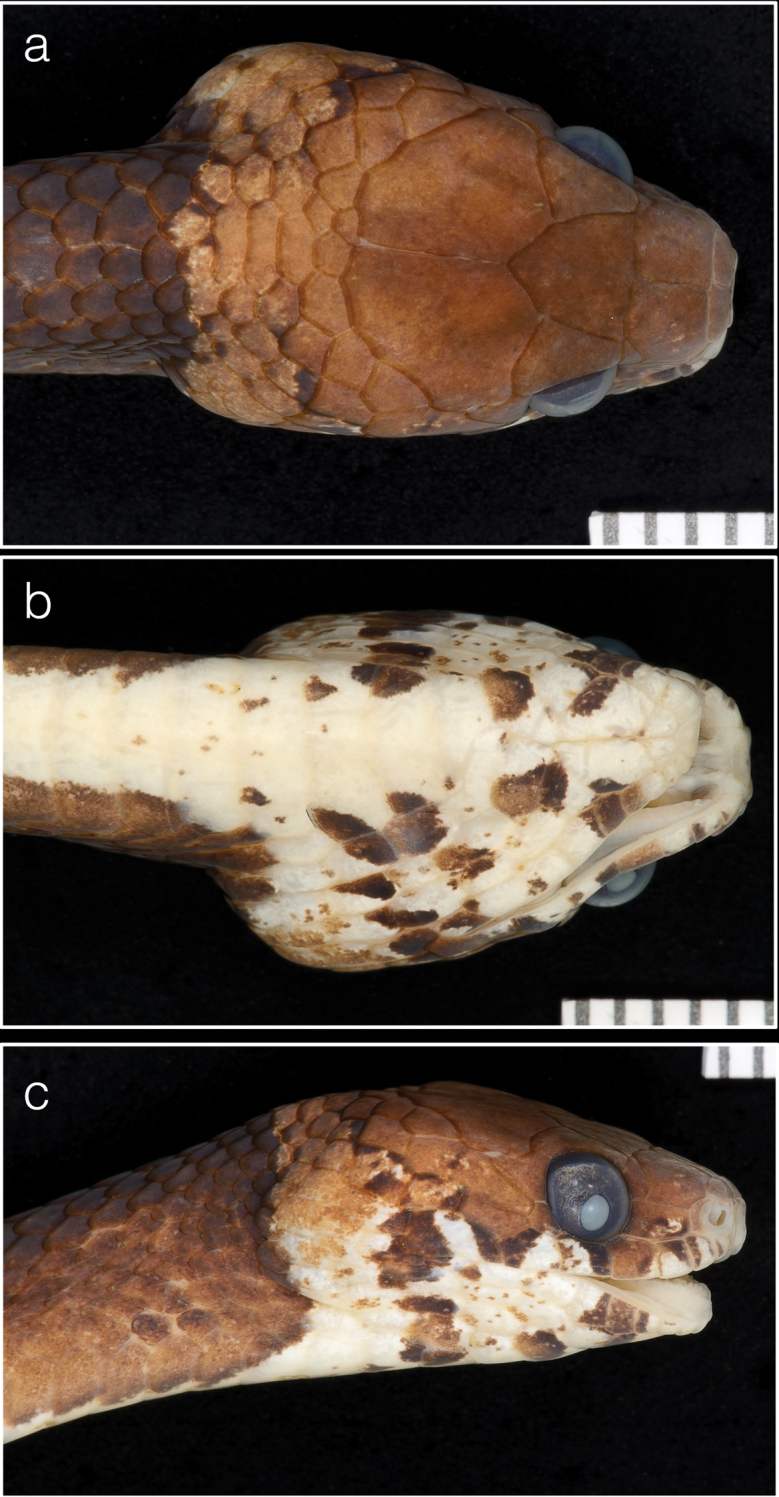
Holotype (USNM 579828) of *Dipsasaparatiritos* sp. nov. showing **a** dorsum of head and **b** chin shields and **c** lateral view. Ruler notches denote mm. Photographs by James Poindexter.

**Figure 5.**
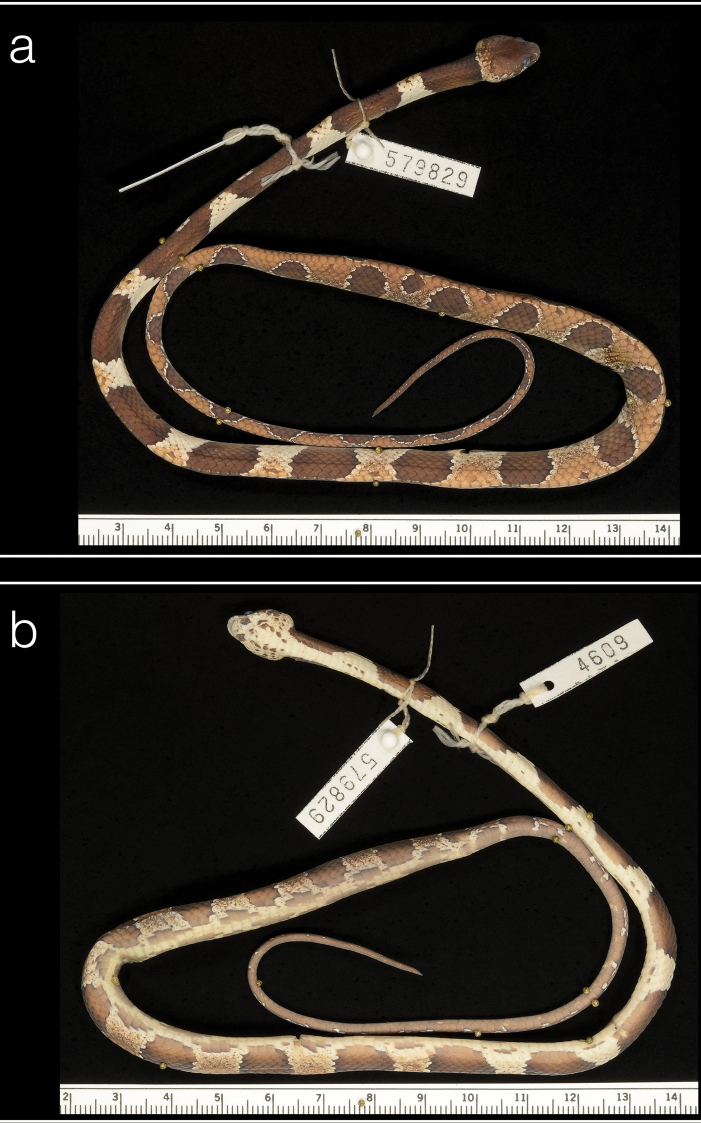
Paratype (USNM 579829) of *Dipsasaparatiritos* sp. nov. showing **a** dorsum and **b** venter. Ruler units in cm. Photographs by James Poindexter.

**Figure 6.**
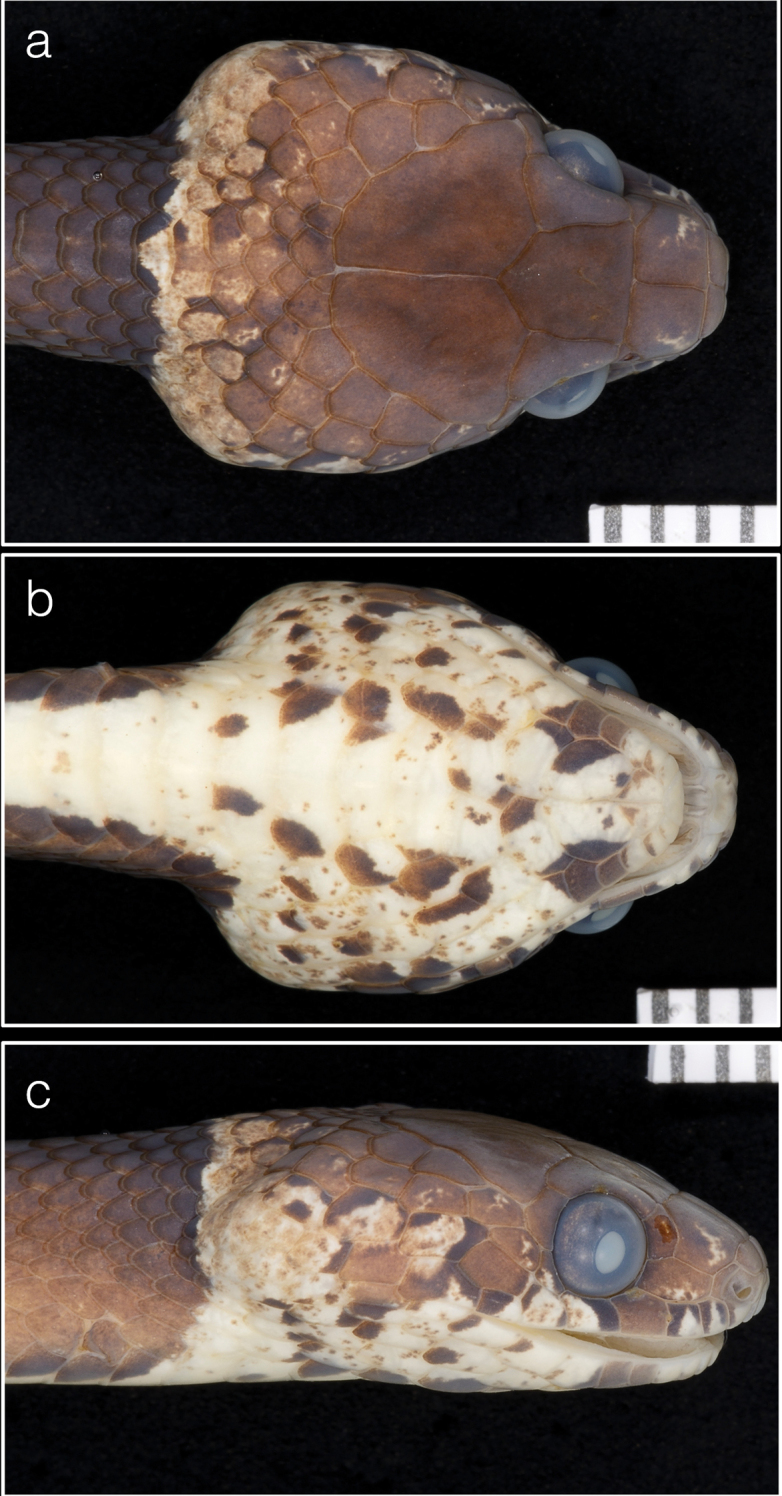
Paratype (USNM 579829) of *Dipsasaparatiritos* sp. nov. showing **a** dorsum of head and **b** chin shields and **c** lateral view. Ruler notches denote mm. Photographs by James Poindexter.

**Figure 7.**
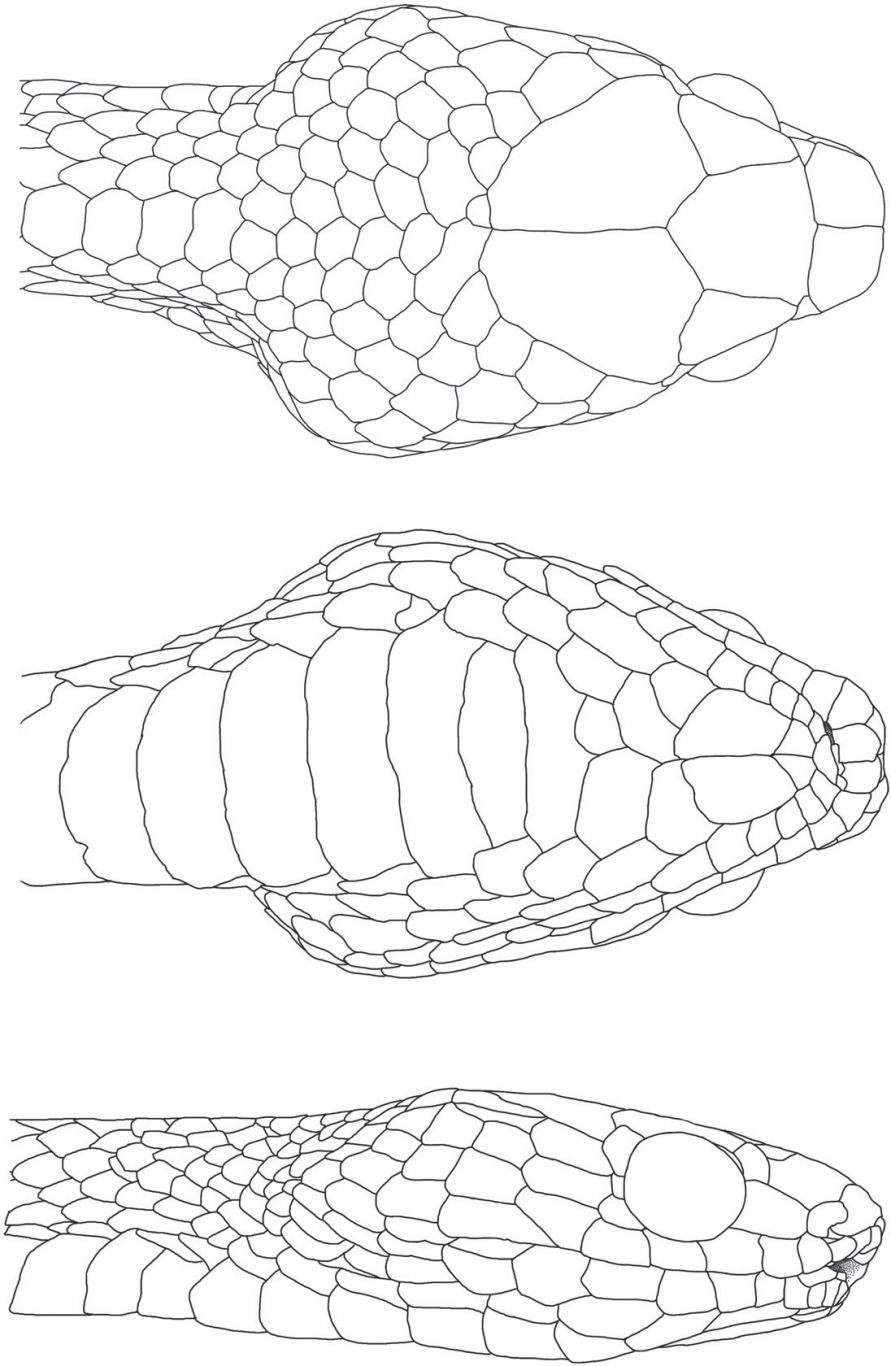
Illustration of the head scales of a *Dipsasaparatiritos* sp. nov. (USNM 579810). Drawings by Shannon Bowley Christensen.

**Figure 8.**
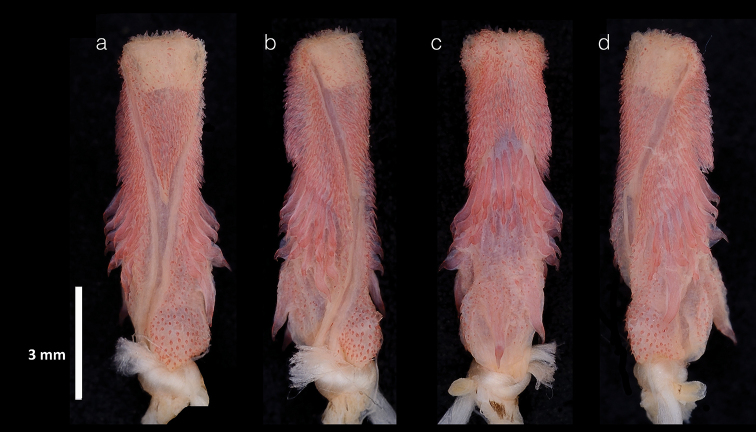
Hemipenes of *Dipsasaparatiritos* sp. nov. USNM 579815 **a** sulcate face **b, d** lateral faces **c** asulcate face. Photographs by James Poindexter.

Figs 2 , 3 , 4 , 5 , 6 , 7 , 8 , Appendix 3 Proposed standard English name: Hidden Snail-eating Snake Proposed standard Spanish name: Caracolera Escondida

##### Type material.

***Holotype*.** Panama • ♀; PNGDOTH, ca. 7.5 km N of El Copé de La Pintada, Coclé Province, 8.670383°N, 80.592343°W, 763 m a.s.l.; 30 Jul 2010; S. Gotte, J. Jacobs, D. Mulcahy and R. Reynolds; USNM 579828 (Biol. Survey Field Series 4608) (Figs 3, 4).

***Paratype*.** Panama • ♀; PNGDOTH, ca. 7.5 km N of El Copé de La Pintada, Coclé Province, 8.670383°N, 80.592343°W, 763 m a.s.l.; 30 Jul 2010; S. Gotte, J. Jacobs, D. Mulcahy and R. Reynolds; USNM 579829 (Biol Survey Field Series 4609) (Figs 5, 6).

##### Diagnosis.

*Dipsasaparatiritos* sp. nov. is placed in the genus *Dipsas* based on phylogenetic evidence (Fig. 1) and the absence of a labial that is noticeably higher than other labials. The species is diagnosed based on the following combination of characters: (1) 15/15/15 smooth dorsals with enlarged vertebral row (1.5–2.4× as wide as adjacent rows); (2) loreal and a preocular in contact with orbit; (3) 7 supralabials with 4^th^ and 5^th^ contacting orbit, 1^st^ supralabial fused with nasal scale; (4) 8–9 infralabials with 3^rd^ to 6^th^ in contact with chin shields, first pair of infralabials not in contact behind symphysial due to presence of two postmentals; (5) 191–196 ventrals in males, 177–197 in females; (6) 122–136 divided subcaudals in males, 111–126 in females; (7) dorsal and ventral color consisting of 17–20 dark brown to black white-bordered body bands (10–12 dorsal scales long anteriorly to 3–5 dorsal scales long posteriorly) separated from each other by white to pale yellow (anteriorly) to pale brown (posteriorly) interspaces measuring 2–6 dorsal scales long, ventral surfaces white with encroachment from the dorsal dark blotches and with smaller blackish marks in-between the blotches, dorsal aspect of head dark reddish brown with small blotches on the labial and temporal scales as well as a pale nuchal collar, throat white with small dark brown to blackish markings, iris pale brown with minute black speckles; (8) 310–465 mm SVL in males, 169–424 mm females; (9) 122–260 mm TL in males, 65–247 mm in females.

##### Description of the holotype.

An adult female; SVL 424 mm; TL 211 mm (49.7% SVL); head broadly distinct from body; head length 13.2 mm (3.10% SVL); head width 7.3 mm (55% head length); snout-orbit distance 3.3 mm; eye diameter 2.5 mm; rostral broader than high, triangular in frontal view, not visible from above; internasals broader than long; prefrontals broader than long and do not enter the orbit; from above, the triangular shape of the top of the preocular is visible; supraocular longer than broad; frontal longer than broad, with a triangular shape in dorsal view; parietals longer than broad; nasal entire and fused with the first supralabial on both sides; loreal longer than high, enters the orbit; one upper preocular; two postoculars; temporals 2+3 left side, 2+2 right side, where the upper primary and secondary scales are fused; 7 supralabials, 4 and 5 contacting orbit (first supralabial is fused with the nasal) symphysial contacting the first pair of chin shields; 9 infralabials; four pairs of irregular chin shields, the first pair is smaller, second pair is longer than broad, the third pair is slightly longer than broad, but its scales are not in contact, the last pair is broader than long. Dorsals smooth in 15-15-15 rows; mid-vertebral scales moderately enlarged; 178 ventral scales; 118 paired subcaudals; cloacal scale single.

In preservative, dorsal ground color of head uniformly brown except for some small dark-brown blotches on the occipital areas; laterals with small pale brown and dark blotches; white supralabials with evident pale brown and dark blotches; ground color of infralabial and gular region cream colored with dark-brown blotches and pale-brown spots; dorsal color of body pale brown with dark-brown blotches and pale interspaces; on the anterior portion of the body the blotches are dark-brown and long (between 10 and 13 scales) contacting the opposite one in the vertebral row, the interspaces are pale brown with small and scarce dark-brown spots on the dorsal, and white on the lateral; on the middle of the body, the dark-brown blotches diminish their length (between 8 and 9 scales), and they lose the dorsal continuation between them in the vertebral row, the interspaces get a pale-brown color with some small dark-brown spots; on the posterior portion, the blotches are shorter (5–7 scales), rounded, and they are margined by a white edge with many small dark-brown spots; ground color of the belly cream-colored, with irregular blotches of different sizes along the ventral line of the interspaces; tail resembles the body in color pattern; body with 16 blotches, and tail with 12. Color in preservative (70% ethanol) similar to color in life.

##### Description of the paratype.

An adult female; SVL 328 mm; TL 170 mm (51.8% SVL); head broadly distinct from body; head length 12.2 (3.7% SVL); head width 6.6mm (54% head length); snout-orbit distance 2.9 mm; eye diameter 2.3 mm; rostral broader than high, triangular in frontal view, not visible from above; internasals, broader than long; prefrontals long as wide, no enter the orbit; from above, the triangular shape of the top of the preocular is visible; supraocular longer than broad; frontal longer than broad, with a triangular shape in dorsal view; parietals longer than broad; nasal entire; loreal longer than high, enters the orbit; one upper preocular; two postoculars; temporals 2+3 left side, 3+4 right side; 8 supralabials, 4 and 5 contacting orbit; symphysial contacting the first pair of chin shields; 9 infralabials; three pairs of irregular chin shields, the first pair is the smaller, second pair is longer than broad; the third pair is slightly broader than long. Dorsals smooth in 15-15-15; vertebral scale moderately enlarged; 183 ventral scales; 124 paired subcaudals; cloacal scale single. In preservative, dorsal ground color of head uniformly brown except for some small dark-brown blotches on the occipital areas; laterals with small blotches pale brown and dark; white supralabials with evident pale brown and dark blotches; ground color of infralabial and gular region cream with dark-brown blotches and pale-brown spots; dorsal color of body pale-brown with dark-brown blotches and pale interspaces; on the anterior and middle portion of the body the blotches are dark-brown and long (12–14 scales) contacting the opposite one in the vertebral row, the interspaces are pale brown with small and scarce dark-brown spots on the dorsal, and white on the lateral; on the posterior portion, the blotches are shorter (between 5 and 7 scales), rounded, they are margined by a white edge with many dark-brown small spots, and they lose the dorsal continuation between them in the vertebral row, the interspaces get a pale-brown color with some small dark-brown spots; ground color of the belly cream, with irregular blotches of different sizes along the ventral line of the interspaces; tail resembles the body; body with 19 blotches, and tail with 15. Color in preservative (70% ethanol) similar to color in life.

##### Referred specimens.

MHCH 2311, juvenile male collected by Sebastian Lotzkat and Andreas Hertz on 18 August 2010 at Cerro Mariposa, Veraguas province, Panama (8.51166°N, 81.12163°W; 940 m), SMF 89551–53, adult males collected by Leonhard Stadler and Nadim Hamad between 8 May and 7 July 2008 at the type locality. SMF 90036, adult male collected by Arcadio Carrizo on 28 July 2008 at Cerro Negro, Veraguas province, Panama (8.56901°N, 81.09894°W; 700 m). SMF 97346, adult male collected by Abel Batista on 25 January 2013 at Donoso, Coclé province, Panama. SMF 89953–54, juvenile and adult of undetermined sex, respectively, collected by Leonhard Stadler and Nadim Hamad on 8 May 2008 at the type locality. SMF 89769, juvenile of undetermined sex collected by Sebastian Lotzkat and Andreas Hertz on 3 April 2009 at Cerro Negro, Veraguas province, Panama (8.56901°N, 81.09894°W; 700 m). MHCH 3123, adult female collected by Marcos Ponce and Roger Morales on 30 May 2018 at Cerro Campana, Panama province, Panama (8.69378°N, 79.92098°W; 730 m).

Additionally, a series of individuals was collected from Parque Nacional General de División Omar Torrijos Herrera between 2006 and 2009 that included 15 females and 12 males. There was variation between sexes and among individuals (Tables 1–3). A summary of the most commonly measured characteristics includes the range of 173–192 ventrals in females (*n* = 11) and 187–191 in males (*n* = 12), subcaudals 116–131 in females (*n* = 13) and 129–136 in males (*n* = 8). All individuals had either 7 or 8 supralabials on both sides (*n* = 26) except one female USNM 579810 with only 6 on the left. Individuals (*n* = 25) had 8 or 9 left infralabials with two individuals having 10. However, the right infralabials ranged from 7–9 with the same individual as above (USNM 579810) having 6 (Fig. 7).

**Table 1.** Measurements of body and head of *Dipsasaparatiritos* to nearest mm. * = Holotype, ** = Paratype.

Catalogue number	Sex	Svl (mm)	Tail length (mm)	Vertebral scale width (mm)	Dorsal scale width (mm)	Eye length (mm)	Rostral to eye length (mm)	Head width (mm)	Head length (mm)
USNM 579820	F	169	65	1.11	0.82	1.98	2.19	4.27	8.90
USNM 579822	F	197	95	1.17	0.78	2.10	1.93	4.29	8.83
USNM 579827	F	205		1.35	0.99	2.19	1.93	4.52	8.80
USNM 579826	F	242	124	1.13	1.04	2.18	2.43	4.86	9.75
USNM 579813	F	265	133	1.17	1.25	2.38	2.25	4.75	9.78
USNM 579825	F	312	175	1.53	1.51	2.51	2.69	4.91	11.85
USNM 579808	F	319	162	1.34	1.46	2.34	2.71	5.00	11.18
USNM 579829**	F	328	170	2.19	1.75	2.34	2.96	6.61	12.28
USNM 579824	F	333	179	1.86	1.70	2.38	2.51	5.09	11.49
USNM 579807	F	346	165	1.64	1.82	2.26	2.99	5.63	12.68
USNM 579823	F	357	189	1.94	1.82	2.54	2.81	5.73	11.98
USNM 579810	F	395	219	2.19	1.65	2.60	2.97	5.97	13.80
USNM 579814	F	400	221	2.06	2.03	2.40	3.54	5.81	13.32
USNM 579809	F	420	221	1.73	2.40	2.58	3.11	6.29	14.00
USNM 579828*	F	424	211	2.70	2.22	2.56	3.31	7.35	13.26
USNM 579816	M	310	122	1.12	1.33	2.47	2.39	5.16	10.57
USNM 579815	M	415	244	1.72	1.83	2.64	3.14	5.91	12.75
USNM 579812	M	420	236	1.63	1.77	2.88	3.00	5.81	12.94
USNM 579819	M	450	251	1.83	2.15	2.83	3.44	5.86	12.06
USNM 579811	M	465	260	1.90	1.69	3.05	3.30	6.05	13.77
USNM 579817	M	465	241	2.18	2.11	2.70	3.27	5.91	13.17

**Table 2.** Scale counts for dorsals, ventrals, labials and loreals, along with dorsal blotch counts for a series of 31 *Dipsasaparatiritos* sp. nov. collected from Parque Nacional General de Division Omar Torrijos Herrera. Also included are available data for specimens collected at other sites. s = single; w = wide loreal; co = contacting the orbit; irr l = irregular loreal. *holotype and **paratype

Catalogue number	Sex	Dorsal scale rows	Ventrals	Sub-caudals	Anal plate	Right supralabials	Left supralabials	Right infralabials	Infralabials contact behind mental	Left infra-labials	Right loreal	Left loreal	Dorsal blotches
SMF 89554	–	15-15-15		116	s	7(4–6)		8					
SMF 89769	Juv	15-15-15	181	181	s	7(4–5)		9					
SMF 89953	Juv	15-15-15	175	110	s	7(4–5)		8					
MHCH 3123	F	15-15-15	194	126	s	7(4–5)		9(2–5)					
USNM 579820	F	15-15-15			s	7–4.5	9–5.6	9	0	8	w co	w co	20
USNM 579822	F	15-15-15		131	s	7–4.5	7–4.5	9	0	9	w co	w co	17
USNM 579827	F	15-15-15			s	7–4.5	7–4.5	9	0	9	w co	w co	18
USNM 579826	F	15-15-15	197	129	s	7–4.5	7–4.5	9	0	9	w co	w co	18
USNM 579813	F	15-15-15	188	125	s	7–4.5	7–4.5	8	0	8	w co	w co	20
USNM 579825	F	15-15-15	177	119	s	7–4.5	7–4.5	9	0	9	w co	w co	19
USNM 579808	F	15-15-15	184	118	s	7–4.5	7–4.5	8	0	8	w co	w co	18
USNM 579829**	F	15-15-15	183	124	s	8–4.5	8–4.5	9	0	9	w co	w co	19
USNM 579824	F	15-15-15		121	s	7–4.5.6	7–4.5.8	8	0	8	w co	w co	19
USNM 579807	F	15-15-15	178	111	s	7–4.5	7–4.5	9	0	9	w co	w co	18
USNM 579823	F	15-15-15	185	122	s	7–4.5	7–4.5	9	0	8	w co	w co	19
USNM 579810	F	15-15-15	182	116	s	7–4.5	6–4.5	8	0	8	w co	w co	
USNM 579814	F	15-15-15	182	124	s	7–4.5	7–4.5	9	0	9	w co	w co	17
USNM 579809	F	15-15-15	180	118	s	7–4.5	7–4.5	9	0	9	w co	w co	18
USNM 579828*	F	15-15-15	178	118	s	7–4.5	7–4.5	9	0	9	w co	w co	16
USNM 579816	M	15-15-15	192		s	7–4.5	7–4.5	9	0		w co	w co	17
USNM 579815	M	15-15-15	196	135	s	7–4.5	8–5.6	9	0	9	w co	w co	17
USNM 579812	M	15-15-15	195	131	s	7–4.5	7–4.5	10	0	9	w co	w co	19
USNM 579819	M	15-15-15	194	136	s	7–4.5	7–4.5	8	0	8	irr l	irr l co	19
USNM 579811	M	15-15-15	195	129	s	7–4.5	7–4.5	9	0	9	w co	w co	18
USNM 579817	M	15-15-15	191		s	8–4.5	7–4.5	9	0	9	w co	w co	19
MHCH 2311	M	15-15-15	194	130	s	7(4–5)		9(3–6)					
SMF 89551	M	15-15-15	191	130	s	7(4–5)		9					
SMF 89552	M	15-15-15	192	122	s	7(4–5)		9/8					
SMF 89553	M	15-15-15	190	130	s	7(4–5)		9					
SMF 90036	M	15-15-15	192	122	s	7(4–5)		9/8					
SMF 97346	M	15-15-15	192		s	–		9					

**Table 3.** Scale counts related to the ocular region of the series of 31 *Dipsasaparatiritos* sp. nov. specimens. upp = upper; low = lower. * = holotype, ** = paratype.

Catalogue number	Sex	Right preocular	Left Preocular	Right presubocular	Left presubocular	Right postocular	Left postocular	Right post-subocular	Left post-subocular
SMF 89554	–					2			
SMF 89769	Juv					3			
SMF 89953	Juv					2			
MHCH 3123	F					2			
USNM 579820	F	1 upp	1 upp	0	0	3	3	0	0
USNM 579822	F	1 upp	1 upp	0	0	2	2	0	0
USNM 579827	F	1 upp	1 upp	0	0	2	2	0	0
USNM 579826	F	1 upp	1 upp	0	0	3	3	0	0
USNM 579813	F	1 upp	1 upp	0	0	2	2	0	0
USNM 579825	F	1 upp	1 upp	0	0	3	3	0	0
USNM 579808	F	1 upp	1 upp	0	0	2	2	0	0
USNM 579829**	F	1 upp	1 upp	0	0	2	2	0	0
USNM 579824	F	1 upp	1 upp	0	0	1 upp/ 1 low	1 upp/ 1 low	0	0
USNM 579807	F	0	0	0	0	2	2	0	0
USNM 579823	F	1 upp	1 upp	0	0	2	2	0	0
USNM 579810	F	1 upp	1 upp	0	0	2	2	0	0
USNM 579814	F	1 upp	1 upp	0	0	2	2	0	0
USNM 579809	F	1 upp	1 upp	0	0	2	1 upp/ 1 low	0	0
USNM 579828*	F	1 upp	1 upp	0	0	2	2	0	0
USNM 579816	M	1 upp	1 upp	0	0	3	3	0	0
USNM 579815	M	1 upp/ 1 low	1 upp/ 1 low	0	0	3	3	0	0
USNM 579812	M	1 upp	1 upp	0	0	2	2	0	0
USNM 579819	M	2	1 upp/ 1 low	0	0	3	2	0	0
USNM 579811	M	1 upp	1 upp	0	0	2	2	0	0
USNM 579817	M	1 upp	1 upp	0	0	2	1 upp/ 1 low	0	0
MHCH 2311	M					4			
SMF 89551	M					2			
SMF 89552	M					3			
SMF 89553	M					3			
SMF 90036	M					3			
SMF 97346	M					3			

##### Hemipenial morphology.

Description based on the hemipenes fully everted, but not completely expanded, for the specimen USNM 579815 (Fig. 8). Distal end of retractor muscle divided, hemipenis unilobed, unicapitate and unicalyculate; capitulum with papillate and spinulate calyces, it covers approximately the distal half of the organ in the sulcate face, and the distal one-third in the asulcate; the inferior capitular edge of the sulcate face is V-shape, and in the asulcate face the capitular arch is present. In both faces, the hemipenial body is covered by a few small spines, and mostly by medium-sized spines which have curved and robust tips. The base of the organ also is covered by dispersed little spinules on both faces; there is not an evident nude pocket, and there are two spines of similar size on the asulcate side. The sulcus spermaticus bifurcates at the base of the capitulum; both branches diverge and extend diagonally oriented, and end at the distal edge of the lateral face of the organ.

##### Comparisons.

*Dipsasaparatiritos* sp. nov. can be distinguished from all other similar or related species by the following combination of characters: 15 dorsal scale rows; one upper preoculars; two or three postoculars; temporals 1+2; seven or eight supralabials, fourth and fifth contacting the orbit; eight or nine infralabials, no infralabials in contact behind mental; vertebral row moderately enlarged; 191–196 ventrals in males, and 177–197 in females; 129–136 subcaudals in males, and 111–131 in females; by the alternating dark brown and tan brown bands running the length of the body, including the tail.

*Dipsasaparatiritos* sp. nov. differs from the majority of its congeners by having the nasal scale fused with the first supralabial, anterior infralabials separated by a pair of (rarely fused) small postmentals, and temporals usually entering the orbit. *Dipsasaparatiritos* sp. nov. shares with the other Central American species of the genus the number of dorsal scales rows (15-15-15), except with *D.gaigeae* Oliver (13-13-13); number of temporals (1+2+ 2); absence of preoculars, except *D.brevifacies* Cope (1, 2 or 3); and number of postoculars (2,3), except *D.temporalis* Werner (3,4). The number of infralabials (9–10) is in the range of all Panamanian species, but the infralabial scales in contact behind mental (0) differs from all species, except with *D.temporalis*. The number of supralabials (7–8) is within the variation found in *D.gaigeae* (7–8), *D.nicholsi* (7–9), *D.temporalis* (6–8), and *D.tenuissima* Taylor (8), but differs from *D.articulata* Cope, *D.bicolor* Günther, *D.brevifacies*, and *D.viguieri* Bocourt (9–10); the supralabials scales in contact with the eye (4–5) also are in the variation found in the other species (Table 4). The vertebral row is enlarged moderately as in *D.nicholsi* and *D.temporalis*, and it different from the other species where it is scarcely enlarged. The number of ventral scales of males and females of *Dipsasaparatiritos* sp. nov.is larger than *D.brevifacies* and *D.gaigeae* and fewer than *D.articulata*, *D.tenuissima* and the males of *D.temporalis*, while overlapping with *D.bicolor*, *D.nicholsi*, *D.viguieri*, and the females of *D.temporalis* (Table 2). The number of subcaudal scales of males and females is larger than *D.brevifacies*, *D.gaigeae*, *D.nicholsi*, and *D.tenuissima*, while overlapping with *D.articulata*, *D.bicolor*, *D.temporalis*, and *D.viguieri* (Table 4).

**Table 4.** Scale counts, measurements and degree of enlargement of the vertebral row of the species of *Dipsas* known to occur in Central America, combining data from the examined specimens listed in Appendix 1 and from references listed in Materials and methods. The values of the ventral and subcaudal counts are minimum and maximum.

	*D.articulata*	*D.bicolor*	*D.brevifacies*	*D.gaigeae*	*D.nicholsi*	*D.aparatiritos*	*D.temporalis*	*D.tenuissima*	*D.viguieri*
**Dorsals**	15-15-15	15-15-15	15-15-15	13-13-13	15-15-15	15-15-15	15-15-15	15-15-15	15-15-15
**Ventrals**	M 198–217 F 195–210	M 195–199 F 185–199	M 167–181 F 166-174	M 162–166 F 163–167	M 192–210 F186–201	M 190–196F 177–197	M 197–208 F 184–192	M 225 F 227	M 196–211 F 190–206
**Subcaudals**	M 115–135 F 108–118	M 129–132 F 111–129	M 71–102 F 69–87	M 64–72 F 53–62	M 81–100 F 84–97	M 122–136F 111–131	M 120–132 F 120–123	M 99 Fno data	M 113–129 F 102–126
**Preoculars**	0	0	1, 2, 3	0	0	0	0	0	0
**Postoculars**	2–3	2–3	3	2	2	2–3	3–4	3	2–3
**Supralabials**	9–10	10	9–10	7–8	7–9	7–8	6–8	8	9–10
**Supralabials contacting eye**	[4,5] [5,6]	[4,5,6,7]	[4,5]	[3,4]	[3,4] [4,5]	[4,5]	[3,4] [4,5]	[4,5]	[4,5] [5,6]
**Infralabials**	10–13	10–12	9–13	7–9	10–13	9–10	8–13	9–10	9–12
**Infralabials in contact**	[1,1]	[1,1]	[2,2]	[1,1]	[1,1] [2,2]	0	0	[1,1]	[1,1]
**Temporals**	2+3+4	1+2+3	2+3+4	2+3+4	2+3+4		2+3+4	2+3+3	2+3+4
**TOL of largest specimen (mm)**	M 715 F 655	M no data F 627	M 596 F 536	M 652 F726	M 861 F 798	M 725 F 713	M 697 F 645	M 554 F 572	M 719 F 547
**Vertebral row**	Scarcely enlarged	Scarcely enlarged	Scarcely enlarged	Not enlarged	Moderately to broadlyenlarged	Moderately enlarged	Moderately to broadlyenlarged	Scarcely enlarged	Scarcely enlarged
**TL / TOL**	M 32% F 31%	M 33% F%	M 30% F 26%	M 23% F 28%	M 25% F 24%	M 35% F 34%	M 38% F 33%	M 29%F no data	M 33%F 30%

The new species is sister to *Dipsastemporalis*, from which it differs on the following characters of coloration and lepidosis. In *D.aparatiritos* sp. nov., the first dorsal band extends far onto the ventrals (restricted to the dorsum or barely entering ventrals in *D.temporalis*) and the posterior body bands form elliptical blotches usually broken along the vertebral line (bands complete over dorsum or elliptical blotches joined along the vertebral line in *D.temporalis*). The color of the anterior interspaces is white or bright pale yellow in *D.aparatiritos* sp. nov. and pale brown in *D.temporalis*. Overall, *D.temporalis* compared to *D.aparatiritos* sp. nov. have a greater number of ventral scales in males (x̄ = 198) vs. (x̄ = 192) and females (x̄ = 192) vs. (x̄ = 184) respectively, although there is overlap in the counts (Table 5, Fig. 10).

**Table 5.** Differences in coloration, scale counts and size between *Dipsastemporalis* and *D.aparatiritos* sp. nov. The range of each continuous variable is from our own sample, Harvey (2008), and Lotzkat (2015). The numbers in parentheses represent the sample size.

Variable	*Dipsastemporalis*		*Dipsasaparatiritos* sp. nov.	
**First dorsal band extends far onto the ventrals**	No		Yes	
**Condition of posterior body bands**	Complete over dorsum or elliptical blotches joined along the vertebral line		Forming elliptical blotches usually broken along the vertebral line	
**Color of anterior interspaces**	Pale brown		White or bright pale yellow	
**Infralabials**	8–9		9–10	
**Sex**	Males (*n* = 5)	Females (*n* = 8)	Males (*n* = 12)	Females (*n* = 16)
**Maximum TOL**	694 mm	630 mm	688 mm	713 mm
**Ventral scales**	183–210	184–203	177–197	190–196
**Subcaudal scales**	112–132	111–134	122–136	111–131

##### Etymology.

The species name is an adjective formed from the Greek word *aparatíritos* (απαρατήρητος), which means unnoticed. The snake has hidden in plain sight for more than forty years at a very well-studied field site for herpetological research. We suggest the common name “Hidden Snail-eater” (“Caracolera Escondida” in Spanish).

##### Distribution.

**Figure 9.**
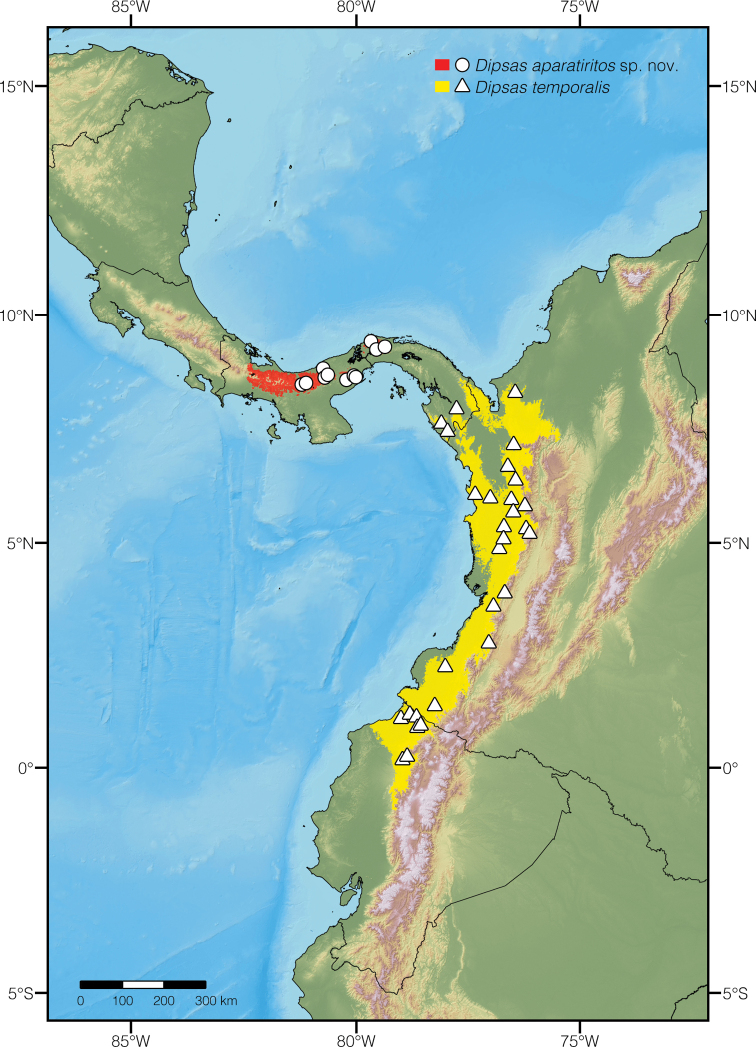
Map of locality data for *Dipsasaparatiritos* sp. nov. (red showing range, circles marking specimens included in this paper) and updated range data for *D.temporalis* (yellow showing range, triangles marking specimens included in this paper).

*Dipsasaparatiritos* sp. nov. is found in both the Atlantic and Pacific slopes of the Cordillera Central in western Panama, with an additional population on the Parque Nacional Chagres. The species occurs over an estimated 9,630 km^2^ area and has been recorded at elevations 597–1002 m above sea level, which makes it the most wide-spread species of *Dipsas* in Panama. A series of individuals were collected from PNGDOTH. This is a mid-elevation, premontane cloud-forest with mature secondary forest and many streams branching from Río Guabal (McCaffery and Lips 2013). The mean annual rainfall is 3500 mm and mean annual temperature range is 19–31 °C (Lips et al. 2006). Two localities (Donoso, Colón province, and Quebrada Las Tres Honeras, Panama province) are in valleys 134–197 m above sea level. Since these localities are much lower in elevation than all other reported localities, it is likely that the specimens collected there (SMF 97346 and MCZ 50214) were actually found in the neighboring mountain ridges (Fig. 9).

**Figure 10.**
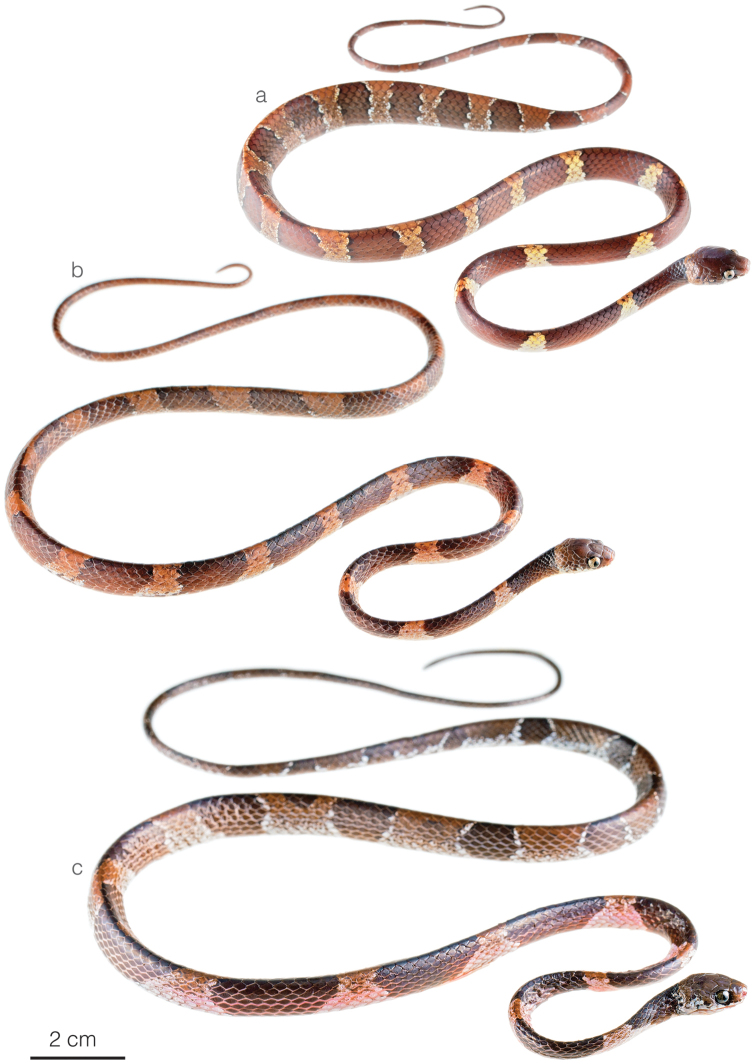
Photographs of species of *Dipsas* previously subsumed under *D.temporalis***a***D.aparatiritos* sp. nov. from Cerro Gaital, Antón, Coclé province, Panama **b***D.temporalis*ZSFQ 5063 from Durango, Esmeraldas province, Ecuador **c***D.temporalis*ZSFQ 5062 from Durango, Esmeraldas province, Ecuador.

##### Natural history notes.

The holotype was encountered at 21:58 h in mature secondary (40+ years) premontane forest on the Atlantic versant, but only ca. 100 m from the Continental Divide. The trail is known as “the old logging road” as described by Myers et al. (2007). The Tropical Amphibian Declines in Streams (TADS) project, which has been working in the area since 1997, refers to the trail as “Rocky Road,” while the park calls it “La Salida” to Sendero La Rana. The snake was elongate and crawling on small tree 0.75 m off the ground. The paratype was encountered at 2159h in mature secondary (40+ years) premontane forest on the Atlantic versant, but only ca. 100 m from the Continental Divide on the same trail as the holotype. The snake was elongate and crawling on small tree 0.75 m off the ground. Lotzkat (2015) found specimens of *Dipsasaparatiritos* sp. nov. foraging at night on vegetation 30–200 cm above the ground. JMR found this species to be more common in forest and along streams rather than around ponds. In PNGDOTH, JMR examined the fecal samples of this species and found that one (2% of the sample) contained the operculum of a snail and 49 (98%) contained oligochaete chaetae.

**Figure 11.**
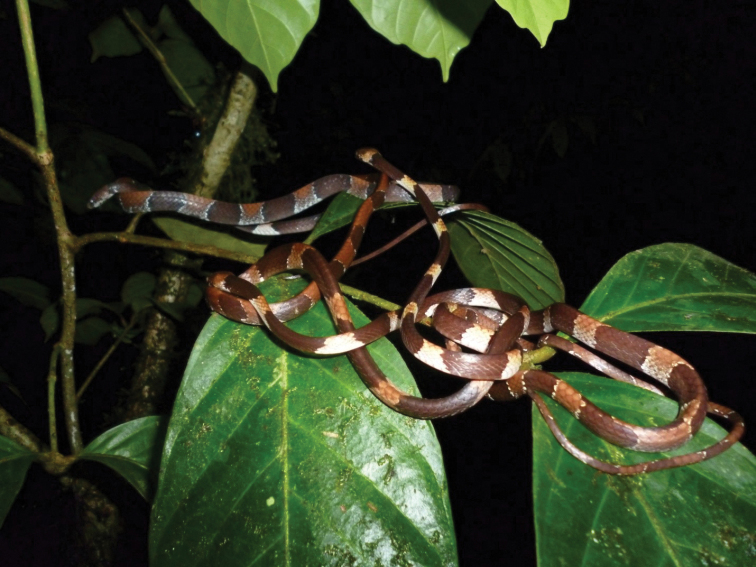
Four individuals of *Dipsasaparatiritos* sp. nov. intertwined on one plant at Parque Nacional General de División Omar Torrijos Herrera. Photograph by Noah Carl.

Despite being a new species, it is relatively common at the PNGDOTH site and has been documented for years, thus providing much data on the natural history. Specimens have been found in vegetation, at times over one meter in height, but at other times just centimeters off the ground where it blended in well with leaf litter, as proven by one individual found on the ground (Fig. 2). Gravid females were found in all months except February, March, October, and December with the highest frequency in June and July (JMR pers. obs.). Females had either one or two ova. Breeding events were not observed, although one night four different *Dipsasaparatiritos* sp. nov. were observed intertwined on a single branch (Fig. 11).

Near the area where the holotype of *Dipsasaparatiritos* sp. nov. was found in PNGDOTH, JMR has recorded the following species of amphibians and reptiles: salamanders including *Oedipinacollaris* (Stejneger, 1907), and *Bolitoglossacolonnea* (Dunn, 1924), frogs, including *Diasporusdiastema* (Cope, 1875), *Espadaranaprosoblepon* (Boettger, 1892), lizards including *Anolishumilis* Peters, 1863, and *Enyalioidesheterolepis* (Bocourt, 1874), and snakes including *Bothropsasper* (Garman, 1883), *Bothriechisschlegelii* (Berthold, 1846), *D.nicholsi*, *Imantodescenchoa* (Linnaeus, 1758), *Oxybelisbrevirostris* (Cope, 1861), *Sibonannulatus*, and *S.nebulatus* (Linnaeus, 1758).

##### Conservation.

We consider *Dipsasaparatiritos* sp. nov. to be included in the Near Threatened category following the IUCN Red List categories and criteria, v. 3.1, second edition (IUCN 2012) because, although the species’ estimated extent of occurrence is less than 10,000 km^2^ and nearly 44% of this area has already been deforested (CATHALAC 2011), the species occurs in at least four major national parks (Santa Fe, PNGDOTH, Altos de Campana, and Chagres) and satellite images show that there is forest connectivity between populations. At PNGDOTH, the occurrence rate of *D.aparatiritos* sp. nov. has actually increased by a factor of three in the period between 2006 and 2012 (Zipkin et al. 2020). Also, the body condition of the individuals in this locality increased following the collapse of amphibian populations due to chytridiomycosis (Zipkin et al. 2020). However, the causes for these changes are enigmatic given that amphibians presumably do not comprise an important part of the diet of this species. The status and trend of other populations should be evaluated carefully given that *D.aparatiritos* sp. nov. is endemic to Panama and probably highly dependent on old-growth forests.

##### Other *Dipsas* species at the site.

In addition to the new species there are two other species of *Dipsas* known from the site: *Dipsasnicholsi* (Myers et al. 2007 [see edit to proof]) and *Dipsasarticulata* (Vecchiet et al. 2014). This adds one more confirmed species, bringing the total to three. Furthermore, also known to occur at the site are at least four species of *Sibon* (*S.argus*, *S.canopy*, *S.longifrenis*, and *S.nebulatus*), which are closely related phylogenetically (Peters 1960; Sheehy 2012) and ecologically (Ray et al. 2011). *Sibonlamari* also may be present at the site (JMR unpubl. data). *Dipsasaparatiritos* sp. nov. was found throughout the general survey area, both on metered-transects and within the adjacent forest between transects.

### ﻿Key to Central American *Dipsas*

**Table:** 

1	Dorsals 13-13-13, loreal longer than high contacting the orbit; preoculars absent; seven supralabials, third and fourth contacting the orbit; 7 or 8 infralabials, one pair in contact behind the mental; vertebral scale not enlarged; ventrals M 162–166, F 163–167; subcaudals M 64–72, F 53–62	***Dipsasgaigeae***
–	Dorsals 15-15-15	**2**
2	Ventrals > 220; square loreal contacting the orbit; one preocular; eight supralabials, fourth and fifth contacting the orbit; 9 or 10 infralabials, one pair in contact behind the mental; vertebral scale slightly enlarged; ventrals M 225, F 227; subcaudals M 99	***Dipsastenuissima***
–	Ventrals < 220	**3**
3	Black horseshoe pattern present on the dorsum of head; irregular or square-shaped loreal contacting the orbit; preoculars absent; 8 or 9 supralabials, fourth and fifth contacting the orbit; 12 infralabials, one pair in contact behind the mental; vertebral scale slightly enlarged; ventrals M192–210, F 186–201; subcaudals M 81–100, F 84–97; beige with dark brown saddles	***Dipsasnicholsi***
–	Lack of black horseshoe pattern on the dorsum of head; typically, dark brown alternating with paler brown or tan; white outline may be present	**4**
4	Alternating brown with pale beige or white with rose/pink/red on white spots of dorsum	**5**
–	Lacking rose/pink/red on white spots of dorsum	**6**
5	Single chin shields; irregular or square shape loreal contacting the orbit; one preocular; 10 supralabials, fourth, fifth, and sixth contacting the orbit; 11 or 12 infralabials, one pair in contact behind the mental; vertebral scale not enlarged; ventrals M 195–199, F 185–199; subcaudals M 129–132, F 111–129	***Dipsasbicolor***
–	Paired chin shields; loreal longer than high or square loreal contacting the orbit; preoculars absent; eight supralabials, fourth and fifth contacting the orbit; 10 or 11 infralabials, one pair in contact behind the mental; vertebral scale slightly enlarged; ventrals M 198–217, F 195–210; subcaudals M 115–135, F 108–118	***Dipsasarticulata***
6	Supralabials 6–8	**7**
–	Supralabials 9	**8**
7	Loreal longer than high contacting the orbit; one preocular; seven or six supralabials, fourth and fifth or third and fourth contacting the orbit; 8–10 infralabials, none in contact behind the mental; vertebral scales slightly enlarged; ventrals M 197–208, F 184–200; subcaudals M 120–132, F 120–123	***Dipsastemporalis***
–	Loreal longer than high contacting the orbit; one preocular; 7 or 8 supralabials, fourth and fifth contacting the orbit; 9 or 10 infralabials, none in contact behind the mental; vertebral scales moderately enlarged; ventrals M 191–196, F 177–197; subcaudals M 129–136, F 111–131, Head pale brown	***Dipsasaparatiritos***
8	Irregular or square shape loreal contacting the orbit; preoculars absent; 9 supralabials, fourth and fifth or sixth contacting the orbit; 9–11 infralabials, one pair in contact behind the mental; vertebral scales slightly enlarged; ventrals M 196–211, F 190–206; subcaudals M 113–129, F 102–126; Head reddish-brown	***Dipsasviguieri***
–	Loreal longer than high contacting the orbit; preoculars one; nine supralabials, fourth and fifth contacting the orbit; 10–12 infralabials, two pairs in contact behind the mental; vertebral scale slight enlarged; ventrals M 167–181, F 166–174; subcaudals M 71–102, F 69–87	***Dipsasbrevifacies***

## ﻿Discussion

In the past decade, a significant number of species have been added to the fauna of Panama, either as range extensions across political borders or as newly described species to science. The former includes *Niniasebae* (Duméril, Bibron, & Duméril, 1854) and *Porthidiumvolcanicum* Solórzano, 1995 in the western part of the country, and *Leptophiscupreus* (Cope, 1868) (Batista and Wilson 2017) and *Micrurusdumerilii* Jan, 1858 (Prairie et al. 2015) in the east. The latter includes dipsadine species such as *Sibonperissostichon* (Köhler et al. 2010) and *S.noalamina* (Lotzkat et al. 2012), along with the colubrine *Tantillaberguidoi* (Batista et al. 2016). Additionally, the number of the very rare *Geophisbellus* Myers, 2003 (Dipsadinae) specimens has increased significantly (Lara et al. 2015 and an additional, complete specimen of *Atractusimperfectus* Myers, 2003 (Dipsadinae) was found (Ray 2017). According to our assessment, the range of *Dipsastemporalis* in Panama has been reduced to the eastern portion of the Darien. However, this species is still currently found in Panama.

Interestingly, *Dipsasaparatiritos* sp. nov. has been known at the PNGDOTH site since the late 1970s when Charles Myers visited and mentioned the potential presence of at least one new species of *Dipsas*. Given how similar it is to the previously documented *D.temporalis*, and that the very rare *D.nicholsi* also was found in this remote area, suggests that other species of *Dipsas* may be found in other isolated, mountainous areas around the country. There is a need for continued research, especially in remote areas, to fully document the serpent fauna of Panama.

*Dipsasaparatiritos* sp. nov. is sister to *D.temporalis*. We have decided to name in our phylogeny the specimen MHUA 14278 as *D.temporalis* following the work of Sheehy (2012) and Arteaga et al. (2018) and to not follow the suggestion by Barros et al. (2012) of identifying the sample as *D.sanctijoannis*. The sample in question was identified before as *D.pratti* (Daza et al. 2009, see GenBank) but Sheehy (2012) incorporated samples of *D.pratti* from the type locality and Venezuela, which clearly represents a different species. Sheehy presents the sample in question as *D.temporalis*. Later, Arteaga et al. (2018) presented a near topotypic sequence of *D.temporalis*, QCAZR5050, from San Lorenzo, Esmeraldas, 866 m. This near topotypic sequence forms a tight clade with the sample in question, MHUA 14278, in their phylogeny. *Dipsastemporalis* is typically a lowland Chocoan species inhabiting from Ecuador to Panama, below 100 m elevation. *Dipsaspratti* is a highland Andean species inhabiting the Cordillera Central and the Cordillera Oriental of Colombia and Venezuela, as shown by Barros et al. (2012). *Dipsassanctijoannis* is a highland species distributed along the Cordillera Occidental and Cordillera Central of Colombia, and known from elevations between 1585 and 2400 (Boulenger 1911; Harvey et al. 2008). The lowest record of *D.sanctijoannis* that we know about is the type, from the town of Pueblo Rico, above the Río San Juan, near the Risaralda-Choco border at 1585 m (Boulenger 1911). Harvey et al. (2008) reports on a specimen of *D.temporalis* from near the type locality and along the San Juan drainage but, from much lower elevation, ca 60 m elevation (USNM 267244). This specimen is less than 90 km away from the type locality of *D.sanctijoannis*. The specimen MHUA 14278, originates from the lowlands of the northwestern branch of the Department of Antioquia, at 233 m elevation. Both the previous phylogenetic analyses and the lowland affinity of *D.temporalis* as compared to the highland *D.pratti* and *D.sanctijoannis* support our taxonomic decision.

Despite being a newly described species, *Dipsasaparatiritos* sp. nov. is quite common at the type locality. Fortunately, this area is a protected national park. Regardless, during the ten years JMR spent studying at the site, there was a reduction in number of park rangers (already very few for such a large, protected area), and there was a decline in the care of the trails near the ranger station. The site was logged in the past and unpermitted collection of rare butterflies was observed at the site, suggesting that other unpermitted collectors could arrive in the future. In 2015, the community began to pave the road leading into the park in an effort to pave to the town of La Rica inside the park boundaries. This advancement will greatly increase the ease with which tourists and poachers alike are able to reach the site. In the past, the site was only accessible with high-clearance four-wheel-drive vehicles. Finally, chytridiomycosis reached the site in 2004, but Ray et al. (2012) showed that *D.aparatiritos* sp. nov. (*Dipsas* sp. in that publication) feeds primarily on oligochaetes. There may be a desire of horticulturists and invertebrate enthusiasts to collect bromeliads where both the bromeligenous oligochaetes and *D.aparatiritos* sp. nov. spend considerable time. It is hoped that the area will remain protected and *D.aparatiritos* sp. nov. can continue to thrive.

## Supplementary Material

XML Treatment for Dipsas aparatiritos

## ﻿Appendix 1

**Specimens examined**

The numbers with an asterisk (*) correspond to holotypes.

*Dipsasaparatiritos* sp. nov. **Panama**, Coclé, Donoso: SMF 97346; El Copé, ca. 5.5 km N of Parque Nacional General de División Omar Torrijos Herrera: USNM 579807–579829; Panamá Oeste, Cerro Campana: MHCH 3123; Veraguas, Cerro Mariposa: MHCH 2311, SMF 89551–53, SMF 89953–54; Cerro Negro: SMF 89769, SMF 90036.

*Dipsasarticulata*. **Nicaragua**, Río San Juan, Elev. 13m, Río Indio Lodge: MVZ 269222–269223; **Panama**, Bocas del Toro: AMNH 124125, Cocuyas de Veragua: ANSP 10113*; Isla Bastimentos, Old Point: USNM 297917; Laguna de Tierra Oscura, 3.7 km S of Tiger Key: USNM 348490–348491; **Costa Rica**, Limón, La Castilla, Lower Reventazon: ANSP 22380; Pandora: UMMZ 125236.

*Dipsasbicolor*. **Honduras**, Gracias a Dios, Bachi Kiamp: USNM 578015; Cabeceras de Río Rus Rus: USNM 559619; Urus Tingni Kiamp: USNM 561921; Warunta Tingni Kiamp: USNM 561922; Olancho, Nueva Esperanza: USNM 559618.

*Dipsasbrevifacies*. **Mexico**, Yucatán: FMNH 20634, 36397, 36401, 36406, USNM 6562; Citilpeck: ANSP 10129.

*Dipsasgaigeae*. **Mexico**, Colima: AMNH 82017; Colima: USNM 160938; Hacienda Paso del Rio/Periquillo: UMMZ 80221*; Jalisco, Barra de Navidad: USNM 196499.

*Dipsasnicholsi*. **Panama**, Canal Zone, Madden Forest Preserve: KU 110310–314; Coclé, Parque Nacional General de División Omar Torrijos Herrera: USNM 579806; Panamá: FMNH 217310, Chagres Village: ANSP 21907.

*Dipsastemporalis*. **Colombia**, Antioquia, unknown: UV-C 5388; Choco, Agua Clara, Río Tamana: USNM 267244; Chocó, Condoto: NHMUK; N slope Alto de Buey: LACM 72747; Valle de Cauca, Tamboral: CPZ-UV 04568; Valle del Cauca, Quebrada la Batea: CZI-R 080; **Panama**, Comarca Emberá-Wounaan, Serranía de Jingurudo: MHCH 2878; Darién, S slope Cerro Cituro, Serrania de Pirre: KU 110301, 110303–304, 110307–309; Ridge between Río Jaqué and Río Imamadó: KU 110295; Panamá, S slope Cerro La Campana: KU 110293; Rancho Frío Field Station: MHCH 2881. **Ecuador**, Esmeraldas, Durango: ZSFQ 5062 and ZSFQ 5063; Junto al Río Chuchubí: QCAZ 5050; 16 km W of Lita: MHNG 2521.083; Tundaloma Lodge: MZUTI 3331.

*Dipsastenuissima*. **Costa Rica**, San José, 15 mi NW San Isidro del General: KU 31961*; **Panama**, Chiriquí, Pto. Armuelles: ANSP 24255; Panama Isthmus, MZUSP 2049.

*Dipsasviguieri*. **Colombia**, Chocó: FMNH 74376; **Panama**, Darién: AMNH 36200; Rio Tuira at Rio Mono: KU 110316; Canal Zone, Madden Forest Preserve: UF 44291, KU 110317; Madden Forest Road, 2.0 mi S. Trans Isthmus Highway: UF 44290; Pipeline Road: UMMZ 155717.

## ﻿Appendix 2

**Table A1.** Primers used in this study, gene, name, direction, sequence (5'–3' direction), and reference.

Primers				Reference
cyt-b	S20596F	(F)	AACCACTCTTGTTAATCAACTACA	Ingrasci 2011
cyt-b	S21790R	(R)	ACCCATGTTTGGTTTACAAAAACAATGCT	Ingrasci 2011
cyt-b	GLUDG	(F)	TGACTTGAARAACCAYCGTTG	Parkinson et al. 2002
cyt-b	AtrCB3	(R)	TGAGAAGTTTTCYGGGTGRTT	Parkinson et al. 2002
ND4	ND4	(F)	CACCTATGACTACCAAAAGCTCATGTAGAAGC	Arévalo et al. 1994
ND4	LEU	(R)	CATTACTTTTACTTGGATTTGCACCA	Arévalo et al. 1994
ND4	605F	(F)	GTCTCCATCTATGACTCCCA	Ingrasci 2011
ND4	L68R	(R)	TACCACTTGGATTTGCACCA	Ingrasci 2011
NT3	NT3-F3	(F)	ATATTTCTGGCTTTTCTCTGTGGC	Noonan and Chippindale 2006
NT3	NT3-R4	(R)	GCGTTTCATAAAAATATTGTTTGACCGG	Noonan and Chippindale 2006
DNAH3	DNAH3-f1	(F)	GGTAAAATGATAGAAGAYTACTG	Townsend et al. 2008
DNAH3	DNAH3-r6	(R)	CTKGAGTTRGAHACAATKATGCCAT	Townsend et al. 2008

## ﻿﻿Appendix 3

**Table A2.** Specimens used in genetic analyses and respective GenBank numbers.

Taxa	Voucher museum number	Field or tissue number	Locality	ND4	cyt-b	NT3	DNAH3
*D.andiana*	Bioparque Amaru RSCDSP 0389	JM 79 (J. M. Daza)	Ecuador: Los Ríos	**JX398453**	**JX398607**	**JX398744**	**JX293843**
*D.aparatiritos*	USMN 579815	JM 664	Panama: Coclé	**JX398476**	**JX398626**		
*D.aparatiritos*	USMN 579814	JM 663	Panama: Coclé	**JX398475**	**JX398625**		
*D.aparatiritos*	USNM HerpTissue 113	JM 758	Panama: Coclé	**JX398477**	**JX398627**	**JX398752**	
*D.aparatiritos*	USMN 579818	JM 795	Panama: Coclé	**JX398478**	**JX398628**	**JX398753**	
*D.articulata*		D161; MSM/ASL at UCR	Costa Rica: Limón	**JX398454**		**JX398740**	
*D.bicolor*		ASL 277 at UCR	Costa Rica: Limón	**JX398455**		**JX398741**	**JX293844**
*D.catesbyi*	DHMECN 11952	ENS 13477	Ecuador: Napo	**JX398456**	**JX398608**		
*D.catesbyi*	UTA R-55949	ENS 12341	Ecuador: Tungurahua	**JX398457**	**JX398609**	**JX398742**	**JX293845**
*D.catesbyi*	UTA R-55974	ENS 12204	Ecuador: Tungurahua	**JX398458**	**JX398610**	**JX398743**	**JX293846**
*D.catesbyi*	KU 214851	WED 57932	Peru: Madre de Dios	EF078537	EF078585		
*D.catesbyi*		WED 59073	Peru: Madre de Dios	**JX398459**	**JX398611**	**JX398745**	**JX293847**
*D.georgejetti*	UTA R-61628	ENS 12817	Ecuador: Manabí	**JX398554**	**JX398694**	**JX398817**	**JX293897**
*D.gracilis*	ICN 12019	RAM 315	Colombia: Cesar	**JX398465**	**JX398615**	**JX398746**	**JX293852**
*D.gracilis*	UTA R-55943	ENS 12671	Ecuador: Esmeraldas	**JX398466**	**JX398616**	**JX398747**	**JX293853**
*D.gracilis*	UTA R-55944	ENS 12672	Ecuador: Esmeraldas	**JX398467**	**JX398617**	**JX398748**	
*D.indica*	KU 204908	WED 56989	Peru: Madre de Dios	**JX398468**	**JX398618**	**JX398734**	**JX293854**
*D.jamespetersi*	Bioparque Amaru RSCDSP 0390	JM 72 (J. M. Daza)	Ecuador: Azuay	**JX398555**	**JX398695**	**JX398818**	**JX293898**
*D.mikanii*		CTMZ 495	Brazil: São Paulo		**JX398693**	**JX398816**	**JX293896**
*D.nicholsi*		JM 812	Panama: Coclé	**JX398469**	**JX398619**		
*D.pavonina*		LSUMZ-H 13989	Brazil: Amazonas	**JX398470**	**JX398620**	**JX398749**	**JX293855**
*D.palmeri*	DHMECN 11954	ENS 12421	Ecuador: Tungurahua	**JX398471**	**JX398621**		
*D.peruana*		LSUMZ-H 1532	Peru: Pasco	**JX398472**	**JX398622**	**JX398750**	**JX293856**
*D.pratti*	MBUCV 6837	TB 149H	Venezuela: Zulia	**JX398473**	**JX398624**	**JX398751**	
*D.pratti*	MHUA 14638		Colombia: Antioquia	**JX398474**	**JX398623**		
*D.temporalis*	MHUA 14278		Colombia: Antioquia	GQ334583	GQ334482	GQ334667	GQ334560
*D.turgida*	LSUMZ 36734	LSUMZ-H 6458	Bolivia: Unknown	**JX398556**	**JX398696**	**JX398819**	**JX293899**
*D.trinitatis*	UWIZM.2011.20.25		Trinidad: Arima	**JX398479**	**JX398629**		
*D.variegata*		D99; Vidal et al. (2000)	French Guiana: Cayenne	**JX398480**	**JX398630**	**JX398737**	**JX293857**
*D.variegata*	MHNLS 18013	ENS 11187	Venezuela: Bolivar	**JX398481**	**JX398631**		
*D.variegata*	UTA R-15772	WWL 3152	Suriname: Marowijne	**JX398482**	**JX398601**	**JX398736**	**JX293858**
*D.vermiculata*	UTA R-55939	ENS 12353	Ecuador: Morona-Santiago	**JX398483**	**JX398632**	**JX398754**	**JX293859**
